# Terpenoid Metabolic Engineering in Photosynthetic Microorganisms

**DOI:** 10.3390/genes9110520

**Published:** 2018-10-23

**Authors:** Konstantinos Vavitsas, Michele Fabris, Claudia E. Vickers

**Affiliations:** 1 Australian Institute of Bioengineering and Nanotechnology, The University of Queensland, Brisbane, QLD 4072, Australia; 2 Climate Change Cluster, University of Technology Sydney, 15 Broadway, Ultimo, NSW 2007, Australia; michele.fabris@uts.edu.au; 3 CSIRO Synthetic Biology Future Science Platform, GPO Box 2583, Brisbane, QLD 4001, Australia

**Keywords:** terpenoids, metabolic engineering, photosynthetic microorganisms, cyanobacteria, diatoms

## Abstract

Terpenoids are a group of natural products that have a variety of essential and non-essential roles in metabolism, in biotic and abiotic interactions, as well as commercial applications such as pharmaceuticals, food additives, and chemical feedstocks. Economic viability for commercial applications is commonly not achievable by using natural source organisms or chemical synthesis. Engineered bio-production in suitable heterologous hosts is often required to achieve commercial viability. However, our poor understanding of regulatory mechanisms and other biochemical processes makes obtaining efficient conversion yields from feedstocks challenging. Moreover, production from carbon dioxide via photosynthesis would significantly increase the environmental and potentially the economic credentials of these processes by disintermediating biomass feedstocks. In this paper, we briefly review terpenoid metabolism, outline some recent advances in terpenoid metabolic engineering, and discuss why photosynthetic unicellular organisms—such as algae and cyanobacteria—might be preferred production platforms for the expression of some of the more challenging terpenoid pathways.

## 1. Introduction

Terpenoids are a chemically diverse class of natural products that are present in all domains of life and number in the tens of thousands of compounds isolated to date (more than 70,000 according to the Dictionary of Natural Products database). The wealth of terpenoid structures provides a variety of roles in central cellular processes (such as electron transport, photosynthesis, membrane fluidity, signaling, and cell wall formation). The greatest terpenoid diversity can be found in plants where they have a myriad of roles including mediating in complex interactions with the biotic and abiotic environment [1,2].

**Figure 1:**
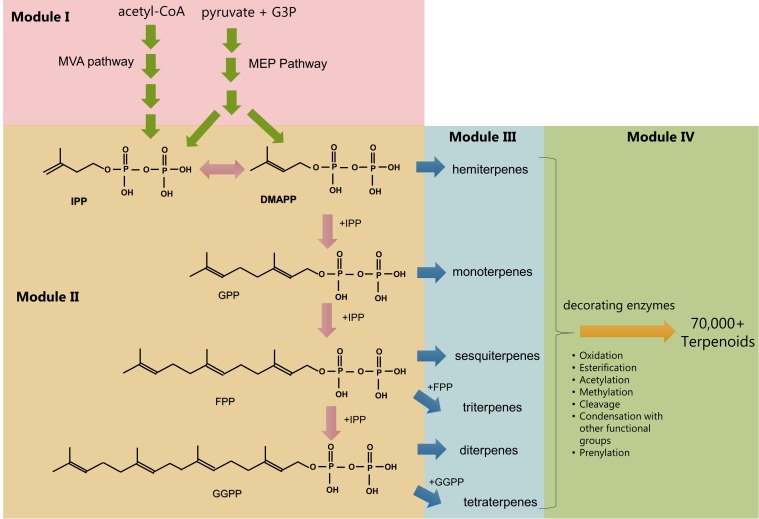
Biosynthesis of terpenoids. The pathways have been conceptually separated into four modules, which is representative of the modularized method many metabolic engineers use to approach isoprenoid pathway engineering. Mevalonate (MVA) and methyl-D-erythritol phosphate (MEP) pathways lead to IPP (isopentenyl pyrophosphate) and DMAPP (dimethylallyl pyrophosphate) (**module I**). Additions of IPP produce higher-order prenyl phosphates (**module II**), dephosphorylation (often coincident with or followed by bond rearrangement and/or cyclisation) to form specialized terpenoid backbones (**module III**), chemical decorations, and other modifications to yield end products. Note that not all end products undergo decorations of the carbon skeleton.

Terpenoid biosynthesis starts with two non-homologous metabolic routes including the mevalonate (MVA) and the methyl-D-erythritol (MEP) pathways. These pathways produce the universal 5-carbon prenyl phosphate precursor molecules isopentenyl pyrophosphate (IPP) and dimethylallyl pyrophosphate (DMAPP) (Figure 1). IPP and DMAPP are condensed to form the C10 prenyl phosphate geranyl pyrophosphate (GPP)—the precursor of monoterpenes. Consecutive addition of IPP units forms farnesyl pyrophosphate (FPP) and geranylgeranyl pyrophosphate (GGPP)—the precursors of sesquiterpenes and diterpenes, respectively (Figure 1). Longer carbon-chain prenyl pyrophosphates are also produced.

The MEP pathway is present in most bacteria, in plastids of photosynthetic organisms, and in some eukaryotic organisms, including pathogens such as the malaria parasite. The MVA pathway appears in archaea and the cytosol of most eukaryotic organisms. Consequently, plants and other eukaryotic organisms—including some algae species—have both pathways present but in different compartments and with distinct roles (e.g., FPP is predominantly supplied by the cytosolic MVA pathway while GPP and GGPP by the chloroplast MEP pathway in plants) [2,3,4]. The prenyl pyrophosphates serve as substrates for specialized terpene synthases that dephosphorylate and rearrange and/or cyclize them by using the energy derived from the pyrophosphate hydrolysis to form the terpenoid skeletons. The last part of the biosynthesis, which delivers the vast terpenoid diversity, is the decoration and modification of the previously mentioned skeleton by enzymes such as cytochrome P450s, acetyl- and methyl- transferases, and cleaving enzymes (Figure 1).

Plant-derived specialized terpenoids are often produced in low quantities or in mixtures of similar compounds (e.g., resins), which makes extraction from natural sources commercially unviable and chemical synthesis very challenging, due to the complexity of the structures of interest [5,6,7]. Consequently, metabolic engineers have turned to heterologous hosts for more efficient production. This, however, presents two fundamental challenges: ensuring a sufficiently high pool of isoprenoid precursors; and the efficient expression and tuning of the heterologous terpenoid pathway to achieve economically viable production.

The industrial workhorses *Escherichia coli* and *Saccharomyces cerevisiae* (yeast) have been the primary target hosts. They are well-characterized heterologous systems with a defined terpenoid background, which facilitates the analytics process and allows the expression of enzymes one by one, or in combinations. This involves the biosynthetic steps of known terpenoids, and even allows the production of new-to-nature compounds [8,9,10,11,12,13]. Key examples of pathways engineered in heterologous hosts using synthetic biology approaches include the production of artemisinic acid [14] and taxadiene [15]—precursors of the anti-malarial artemisinin and cancer chemotherapeutic taxol, respectively. There have been many successful engineering studies that have increased the range of produced compounds and our understanding on how to engineer terpenoid pathways [16,17,18,19,20].

In this review, we summarize key advances that used metabolic engineering to both rewire the host’s primary metabolism to maximize the production of isoprenoid precursors and to enable the production of the desired terpenoid product in tractable, well studied hosts *E. coli* and yeast. We then explore advances in engineering photosynthetic microorganisms and discuss their potential as alternative production platforms for heterologous terpenoids.

## 2. Pathway Modules in Terpenoid Biosynthesis

Metabolic engineering is the application of systems and synthetic biology approaches to engineering cells for the production of industrially useful biochemicals. These factories can be designed and engineered for optimal resource allocation towards the desired pathway. Terpenoid precursor and biosynthetic pathways are well characterized in different organisms and have been manipulated in a multitude of studies, which renders them model pathways for metabolic engineering. The biosynthesis of terpenoids has a distinctive modular structure, which makes their pathways particularly suited to synthetic biology-based engineering approaches: a natural grouping of biochemical reaction types creates four distinct modules that can be independently manipulated (Figure 1). Pathway modularization is useful in combinatorial engineering approaches [11,21,22] and is useful as a concept guiding engineering studies.

### 2.1. Module I: Redirection of Resources Towards Isopentenyl Pyrophosphate and Dimethylallyl Pyrophosphate

Production hosts commonly have insufficient availability of the C5 prenyl pyrophosphate precursors IPP and DMAPP to provide an adequate flux to the heterologous pathways. Successful examples of terpenoid production hosts include maximization of the metabolic flux towards the formation of IPP and DMAPP through the overexpression or manipulation of genes of the endogenous MVA and MEP pathways as well as the insertion of the MEP pathway in organisms with only an MVA pathway to supplement flux [23,24,25,26,27,28]. The latter strategy has the additional objective of overcoming endogenous flux-constraining mechanisms as well as introducing emergent synergistic effects. One interesting study aimed at the production of isoprene pinpointed that, when both the MVA and MEP pathways are co-expressed in *E. coli*, they work in synergy and both have a higher flux when compared to having only one pathway present [27,29].

In the MEP pathway, the first two steps catalyzed by deoxy-xylulose 5-phosphate synthase and deoxy-xylulose 5-phosphate reductoisomerase (DXS and DXR, respectively) as well as the isomerization of C5 prenyl phosphates by the isopentenyl-diphosphate delta-isomerase (IDI) (Figure 1) are rate limiting and must be overexpressed for a high terpenoid yield [15,30,31]. The MVA pathway is limited by the first catalytic steps—from acetyl-coA to mevalonate—and in particular by the 3-hydroxy-3-methyl-glutaryl-coenzyme A (HMG-CoA) reductase [32,33].

In the presence of the MVA/MEP pathway overexpression/augmentation, over-accumulation of prenyl phosphates is problematic and causes cellular toxicity [34]. For this reason, a balance between flux through the DMAPP/IPP synthetic pathways and consumption of prenyl phosphates is required. A strong sink “pull” approach is commonly required to compensate for the upstream pathway flux augmentation.

### 2.2. Module II: Prenyl Phosphate Metabolism

Different classes of terpenoids are produced from prenyl phosphates with different chain-length carbon skeletons (Figure 1). Hemiterpenes are produced from the C5 prenyl phosphates with the best known example being isoprene, which is derived from the DMAPP. The sequential addition of IPP units to DMAPP generates the longer prenyl phosphate skeletons—namely the precursors GPP (C10 terpenoids), FPP (C15 and C30 terpenoids), and GGPP (C20 and C40 terpenoids) (Figure 1).

Both *E. coli* and *S. cerevisiae* preferentially produce FPP, which is a key precursor for the primary isoprenoids produced by these organisms (e.g., sterols in yeast, dolichols in *E. coli*). As a consequence, sesquiterpene (C15) production in these organisms has yielded relatively high titers especially in yeast [14,35]. The competition for the FPP by sterol catabolism must be alleviated and squalene synthase, which consumes FPP and is the first committed step of sterol biosynthesis, has been targeted for down-regulation for this reason [16,36,37,38].

Directing productivity towards the C10 and C20 branches faces different challenges. In *S. cerevisiae* and *E. coli*, the primary prenyl transferase converts GPP immediately to FPP by the addition of a second IPP unit without releasing GPP from the active site under normal conditions [39]. This means that there is a very low in vivo pool of GPP for the production of monoterpenes. In yeast, an FPP synthase (FPPS) engineered to exclude FPP from the active site has been used to increase GPP and monoterpene production [40,41]. An alternative approach is the use of a degradation tag on FPPS to minimize competition and limit flux towards FPP, which enhances monoterpene production [41]. This approach had to be paired with the replacement of the FPPS promoter by the sterol-responsive promoter of squalene epoxidase to ensure sufficient activity of this essential enzyme [41].

Accumulation of GGPP requires an uninhibited flux through GPP and FPP and usually a strong “metabolic pull” strategy is used by overexpressing GGPP synthase [15,42,43]. A more elaborate strategy was also used in *S. cerevisiae* whereby the GPP/FPP synthase was engineered to expand its substrate binding site, which allows it to add an IPP unit to catalyze GGPP formation [44]. This allowed for the enhanced heterologous diterpenoid production.

### 2.3. Modules III and IV: Terpene Synthases, Skeleton Decorations, and Further Modifications

The next two modules comprise the specific pathway for each target compound and are where the true terpenoid diversity is unlocked. Terpene synthases (Module III) form a wide variety of carbon skeletons. These skeletons are the substrates of the decorating enzymes of Module IV. This chemistry is highly complex and results in massive diversity. In this case, we outline generic information to provide (a) a more complete overview of terpenoid biosynthesis and (b) context for the following section on terpenoid production by photosynthetic organisms.

Terpene synthases are often multimeric and slow enzymes [45]. These features make them challenging to use in heterologous production. The solution commonly employed is massive overexpression, which, while it can increase catalytic activity, also creates problems such as inclusion of body formation and loss of activity [46,47,48]. In plants, terpene synthases range in number from one (in the case of the moss *Physcomitrella patens*) to over a hundred per genome [49]. They are thought to have evolved by descent from a single gene resembling the kaurene synthase found in moss and they are notoriously promiscuous enzymes with a high level of substrates and product flexibility [49,50]. While undesirable in many cases (where a specific product is sought), these properties offer opportunities. Terpene synthases can be combined in heterologous systems to produce many different compounds including compounds which are new to nature and have desirable industrial properties [11,12,22,51].

Decorating enzymes—that alter the basic carbon skeleton—are usually the final step of terpenoid biosynthesis. Various enzymes such as oxidative enzymes (especially cytochromes P450, monooxygenases that add hydroxyl groups with a high degree of stereospecificity), methyltransferases, acyltransferases, and prenyltransferases diversify the already numerous terpene skeletons [1]. The cytochromes P450 are one of the keys to unlocking the large terpenoid diversity [5,52]. Cytochrome P450s due to their unique catalytic mechanism are able to introduce hydroxyl groups in a very precise manner even in substrates with a large amount of chiral carbons [53]. They require two electrons per reaction, which is usually provided by NADPH and via a specific cytochrome P450 redox partner even though several other electron carriers can drive the reactions forward [54,55]. Cytochrome P450s are arguably challenging enzymes to engineer in heterologous hosts due to the need for redox power in the form of NADPH, cofactor availability—P450s contain heme molecules—and poor expression of eukaryotic P450s in bacteria [56,57].

### 2.4. General Considerations Spanning Across Nodes

As discussed above, precise control of the heterologous biosynthetic pathways is often crucial to increase production. One way to help balance pathway flux across metabolic modules is to apply metabolite biosensors linked to feedback control mechanisms. Such a strategy can tie enzyme production (for example, terpene synthases) with internal metabolite pools (such as the enzyme’s substrate or a key precursor molecule), which enables the production of the relevant enzyme exactly when required. Moreover, biosensors can be used to implement directed evolution methodologies, which connect compound concentration with growth or other responses. Two successful examples are the use of an NADPH sensor for neurosporene production enhancement and an IPP sensor for lycopene production enhancement [58,59]. Ng and co-workers explored the notion that terpenoid production relies heavily on NADPH availability (and thus regeneration). They generated an NADPH fluorescent sensor to screen many *E. coli* strain variants, selecting the ones with higher NADPH regeneration rates [58]. This allowed them to improve 25-fold the production titers of the carotenoid neurosporene. Chou and Keasling constructed an elaborate IPP-controlled genetic circuit: low IPP causes high mutagenesis rate while, as the IPP concentration increases, the mutation rate drops [59]. Since this is tied to a fluorescent reporter, the researchers could select the strains with lower fluorescence (which had higher IPP production rates) and confirm that these strains could accumulate significantly more lycopene.

Flux reporters such as the carotenoids lycopene and beta-carotene are commonly used as indicators of the terpenoid yield since they give a colored phenotype that can be assessed by absorption or fluorescence. This has been exploited in studies to identify elements such as transcription factors that have an effect on production, coupled with directed evolution or high throughput engineering approaches [60,61,62]. There are, however, some problems with using lycopene as a flux reporter in that changes in the cellular redox status result in oxidation of lycopene and loss of color—thereby interfering with its effectiveness as a pathway flux reporter [62]. In addition, accumulation is affected by prenyl phosphate metabolism influencers and may not relate directly to a core isoprenoid (MEP/MVA) pathway flux. These considerations can render lycopene a poor reporter of pathway flux in some cases. Alternative reporters such as isoprene may be more useful, but assays are not as high throughput or as facile as the colored products.

It should be noted that MEP and MVA pathways both consume reducing power by using NADPH and NADH, respectively. The availability of these redox partners as well as haem molecules (serving as enzyme cofactors) and the precursors acetyl-coA (for the MVA pathway) and pyruvate and glyceraldehyde 3-phosphate (of the MEP pathway) are limiting steps. Therefore, the introduction of pathways that enable the recycling of cofactors or increase their availability have been used successfully to increase terpenoid production [63,64,65,66,67].

Often, it is not easy to optimize all four nodes and produce a terpenoid of interest in a single organism. The use of dual-organism production systems is an interesting alternative. An example has been implemented in the production of oxygenated products of taxadiene. *E. coli* was optimized to produce taxadiene, which was oxygenated by a yeast expressing taxadiene 5α-hydroxylase (a cytochrome P450) and the P450 reductase [68]. This microbial consortium approach shows that different organisms are needed to optimally catalyze different reactions where the enzymes require bespoken environments provided by different hosts.

## 3. Photosynthetic Microorganisms as Terpenoid Production Hosts

The heterologous production of terpenes requires the expression of foreign enzymes and often entire metabolic pathways. *E. coli* and *S. cerevisiae* are currently the main hosts for terpenoid production chosen for their high growth rates, relative ease of engineering, scalability, bioprocess technology, and—in particular—for the advanced knowledge available on their metabolism and genetic resources. However, these two organisms have not evolved to produce a wide variety of complex terpenoid metabolites.

**Figure 2:**
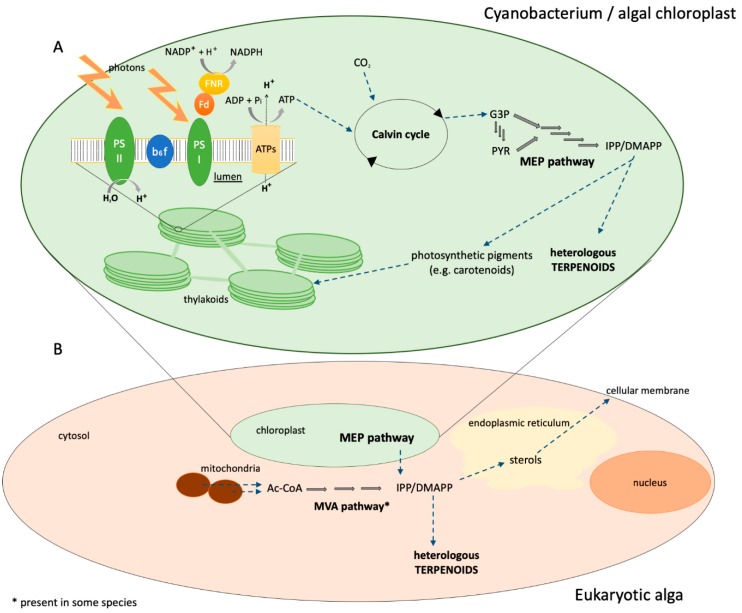
Schematic representation of photosynthetic organisms as heterologous terpenoid biofactories. (**A**) In cyanobacteria and in algal chloroplasts, photosynthesis provides ATP and NADPH, which are, in turn, used to fix carbon dioxide. Glyceraldehyde 3-phosphate (G3P) links carbon fixation with the rest of metabolism. Pyruvate (PYR) is produced from G3P via glycolysis and these two metabolites are the precursors for the MEP pathway. (**B**) In eukaryotic algae, mitochondria produce acetyl-CoA (Ac-CoA) from which the MVA pathway is initiated and IPP/DMAPP are produced. Chloroplasts also contribute to the terpenoid precursor pool. Metabolite exchange between cellular compartments, which is not yet fully elucidated, makes terpenoid metabolism more complex.

Photosynthetic organisms produce a much greater diversity of isoprenoids and use them for many more metabolic processes than yeast and *E. coli*. Theses isoprenoids are also required in higher amounts to satisfy metabolic demands for the production of photosynthetic pigments, sterols, isoprene, and secondary metabolites. Extensive engineering is required to get reasonable flux to isoprenoids in both yeast and *E. coli*, which suggests that isoprenoid metabolism is relatively inefficient in these organisms [16,69]. Conversely, photosynthetic organisms dedicate a significant—albeit small when compared to the reactions of the central carbon metabolism—amount of metabolic resources to terpenoids to provide the production requirements for chlorophyll, carotenoids, and other photosynthetic pigments (Figure 2). While this means that the potential for interference of native metabolism is higher, it also indicates a much greater flux potential for isoprenoid pathways in photosynthetic organisms. The production of massive amounts of isoprene by numerous photosynthetic land plants and microalgae also points to a higher native flux capacity [70]. Moreover, photosynthetic organisms have an increased NADPH content, use of CO_2_ as a feedstock (rather than secrete it in the atmosphere), and offer the ability to directly link photosynthesis with heterologous metabolite production via electron transfer proteins [54,71]. Cyanobacteria and eukaryotic microalgae combine photosynthetic growth with simple cellular organization and, in model species, straightforward genetic manipulation methodologies. In the following sections, we review terpenoid engineering in these photosynthetic micro-organisms (Table 1).

### 3.1. Cyanobacteria

Cyanobacteria are receiving increased attention as potential photosynthetic production hosts due to their simple cellular organization and their metabolic repertoire, which is characterized by high plasticity [71,86,87,88]. The toolbox of synthetic and systems biology resources for metabolic engineering is rapidly increasing and now includes genome-scale metabolic models [89,90], transposon libraries [91], CRISPR tools [92,93,94], and several genetic synthetic biology parts [71,95,96]. Although generation of analysable transgenic lines takes longer than yeast and *E. coli*, they are attractive production hosts due to their photosynthetic growth and relatively simple cellular organization.

The first study in cyanobacterial terpenoid engineering involved the production of the hemiterpene isoprene in *Synechocystis* sp. PCC 6803 [97]. The relatively low initial yield increased fivefold (from 0.05 mg g^−1^ to 0.25 mg g^−1^) with the heterologous expression of the MVA pathway [24]. It is interesting to note that the same strategy yielded a much greater, 400-fold increase in isoprene production in *E. coli* [23]. This highlights differential regulation of isoprenoid metabolism between the two types of organism. It is likely that the large increase in *E. coli* is due to a low innate pathway flux in this organism relative to *Synechocystis* sp. PCC 6803, which provides a larger dynamic range for improvement. It may also reflect availability of central carbon intermediates to feed into the pathways, differential enzyme behavior in the different organisms, or different availability of DMAPP for isoprene synthase. Isoprene production is limited by the amount of the isoprene synthase expressed. Fusion of the isoprene synthase with the c-phycocyanin beta subunit, which is a highly expressed protein, provided a yield increase to 5.4 mg g^−1^ [98]. Overexpressing the IPP isomerase to help balance availability of DMAPP conferred a further improvement to 12.3 mg g^−1^ [72].

A different approach was used in isoprene production in *Synechococcus elongatus* PCC 7942, which resulted in the accumulation of 1.26 g L^−1^ (approximately 540 mg g^−1^) isoprene after three weeks of growth [73]. This is an impressive outcome in engineered cyanobacteria and by far the highest titres to date. In this work, Gao and co-workers focused on enhancing the flux of the MEP pathway by using targeted metabolite analysis to identify bottlenecks and combining several terpenoid enhancement strategies. A similar approach was implemented in *Synechocystis* sp. PCC 6803 by studying the effect of overexpressing the MEP pathway enzymes in isoprene production [99]. Many (but not all) of the MEP enzymes (DXS, DXR, ispD, ispE, ispF, ispH) as well as the IDI isomerase had a positive effect. The most dramatic effect though was observed when more efficient isoprene synthases were used, which highlights that the true bottleneck lies in the terpene synthase activity.

An early effort to engineer monoterpenoid production in *Synechocystis* sp. PCC 6803, which expresses the codon-optimized beta-phellandrene synthase from *Lavandula angustifolia* and reports 50 μg beta-phellandrene L^−1^ [100]. Fusion of the beta-phellandrene synthase with the c-phycocyanin beta subunit resulted in beta-phellandrene accumulation at 3.2 mg g^−1^ [74]. Davies and co-workers explored the production of the monoterpene limonene and the sesquiterpenes bisabolene in *Synechococcus* sp. PCC 7002, which achieves yields of 4.0 mg L^−1^ and 0.6 mg L^−1^, respectively [75]. An increased limonene yield (6.7 mg L^−1^) was achieved in *Synechocystis* sp. PCC 6803 when two enzymes of the pentose phosphate cycle (the ribose 5-phosphate isomerase and ribulose 5-phosphate 3-epimerase) together with a GPP synthase to increase precursor availability were overexpressed [76].

Sesquiterpenoid production was explored in *Anabaena* sp. PCC 7120 where farnesene titres reached 0.08 mg g^−1^ after two weeks of growth [77]. The highest sesquiterpenoid production reported so far was in *Synechococcus elongatus* 7942, which reached 19.8 mg L^−1^ amorphadiene [78]. The authors achieved this by heterologously overexpressing the *E. coli* DXS, FPP synthase, and the IPP isomerase. In *Synechocystis* sp. PCC 6803, squalene, a triterpenoid derived from FPP by the action of squalene synthase, accumulated to approximately 1.2 mg g^−1^ when the downstream squalene hopene cyclase was knocked out [79]. Again, the overexpression of DXS, FPP synthase, and the IPP isomerase resulted in almost 10-fold productivity improvement [78]. The overexpression of the MEP enzymes was implemented in a recent alpha-phellandrine production work in *Synechococcus elongatus* 7942, which reached 4.6 mg L^−1^ alpha-farnesene after seven days of cultivation [80].

Diterpenoid expression in cyanobacteria is less studied. Geranyllinalool, which is a diterpene alcohol present in tobacco, was produced in *Synechocystis* sp. PCC 6803 with reported titres of 0.36 mg g^−1^ [81]. Englund and co-workers produced 13R-manoyl oxide and reached titres of 0.75 mg L^−1^ [101]. Manoyl oxide yield improved to 2 mg L^−1^ by codon-optimizing the terpene synthases and expressing them in a self-replicating vector under a strong promoter [82].

As summarized above, production of terpenoids in cyanobacteria has not yielded very high titers so far—high µg L^−1^ (or µg g^−1^) to low mg L^−1^ (or mg g^−1^)—with the notable exception of isoprene production in *Synechococcus elongatus* (>1 g/L [73]). In an interesting comparison of the isoprene, monoterpene, and sesquiterpenoid yields reported in *E. coli* and cyanobacteria (normalized to total carbon), Ko et al. note that, in most cases, the cyanobacterial titres are disappointingly low in comparison [102]. The effects of terpenoid engineering on native metabolism are also important to study. In the isoprene production strain developed by Gao et al. [73], the carbon fixation rate of *S. elongatus* almost doubled. An increased biomass productivity caused by a sink effect has been observed in other metabolic engineering studies (e.g., [103]). Cyanobacteria seem to tolerate the redirection of carbon towards heterologous terpenoid production and increase the flux towards the MEP pathway to compensate for the extra resources consumed [82]. The MEP pathway provides precursors for carotenoids and chlorophyll, which potentially render any significant imbalance due to heterologous terpenoid production unfavorable for growth. Nevertheless, negative effects such as growth retardation and reduction in oxygen evolution have been observed routinely [78,82,104]. An interesting observation is that knocking out competing pathways does not seem to improve terpenoid production, which is observed when glycogen synthase and squalene hopene cyclase were knocked out in an attempt to enhance limonene and manoyl oxide production, respectively [75,101].

Many enzymes involved in terpenoid biosynthesis both in Module I (MEP/MVA pathway) and in Module IV (decorations) require electron donors as redox partners. Therefore, the redox availability increases flexibility and makes redox enzyme expression more straightforward. One example is the expression of cytochrome P450s in cyanobacteria by using ferredoxin as a direct link to a photosynthetic redox power. This strategy not only results in active enzymes but also may increase overall photosynthetic capacity [105,106,107]. Cyanobacteria seems to have efficient regulation mechanisms that buffer the effects of metabolic perturbations to maintain homeostasis in primary metabolism. This can be both advantageous (as important native branching pathways are not affected by the heterologous ones) but may also hinder metabolic engineering [82]. Another noteworthy observation is that different cyanobacterial strains behave very differently. It is important to keep in mind that, despite their similar morphology, cyanobacterial species are genetically divergent and this may result in a varied behavior in terpenoid production. The cyanobacterial species diversified around 2.5–2.1 billion years ago [108], which means that, in practice, *Synechococcus elongatus* and *Nostoc* sp. PCC 7129 are more distant phylogenetically to each other than humans are to plants. This may explain significantly different production profiles in the presence of engineering between different species.

These studies highlight that, while native MEP pathway flux in cyanobacteria may be relatively high, engineering heterologous terpenoid production still faces challenges in these organisms. Competition with native terpenoid production as well as a relatively poor understanding of regulatory mechanisms are two key challenges that require further attention. Despite this, the ability of these organisms to tolerate and compensate for heterologous terpenoid production is extremely promising.

### 3.2. Eukaryotic Algae

Microalgae are unicellular, (mostly) photosynthetic eukaryotes and form an enormously diverse group that spans across several branches of the eukaryotic tree of life. Because of their huge diversity, this group of organisms is largely unexplored. However, their terpenoid repertoire is estimated to be vast and diverse [109]. Microalgae are promising candidates as production platforms for high-value product manufacturing in light of several intrinsic advantages. They are eukaryotic. Therefore, they are capable of carrying out complex biological and biochemical functions. They are photosynthetic and, thus, have inexpensive growth requirements. In most cases, algae are resistant to stresses usually associated with industrial cultivation and they are often suitable for industrial production scale-up.

Recent advances in sequencing technologies, metabolomics, and proteomics have paved the way to the metabolic characterization at the genetic level of representative microalgal species. The development of increasingly efficient genetic toolboxes for model algal species [110,111,112] allows targeted genetic manipulations for metabolic characterization and gene discovery as well as metabolic engineering. In addition, the recent development of metabolic networks [113,114,115] and genome-scale metabolic models of the main model species [116,117,118,119,120,121] have placed those species under the spotlight as promising next generation, solar-powered, cell-sized biofactories. Among many applications, some microalgae species are currently emerging as attractive alternative platforms for the heterologous production of industrially relevant terpenoids.

#### 3.2.1. *Chlamydomonas reinhardtii*

The green alga *C. reinhardtii* is historically the longest and most widely used model organism for algal genetics. Efforts in engineering this species have provided proof-of-concept for its suitability as a single-celled factory for the synthesis of many bio-products from recombinant therapeuticenzymes to small molecules. The generation of the mutant *C. reinhardtii* strains that display to consistently high levels of transgene expression such as UMV4 and UMV11 [122], which greatly facilitated metabolic engineering in this algal species. Its isoprenoid metabolism is based on solely the MEP pathway to provide precursors for photosynthetic pigments and sterols and no complex terpenes or terpene-based secondary metabolites have been reported to date. Recent work has shown the potential of using *C. reinhardtii* to produce the sesquiterpenoid patchoulol by expressing a plant patchoulol synthase gene [83], which is followed by the evaluation of terpene yield in different growth conditions. This reaches the maximum titer of 1.03 mg L^−1^ in photoautotrophic growth. By comparing previously published sesquiterpenoid yield in cyanobacteria with those of *C. reinhardtii*, Lauersen et al. hypothesized that *C. reinhardtii* might have a relatively larger availability of farnesyl pyrophosphate [75,83]. However, this work highlighted the availability of prenylphosphate precursors as bottleneck for the heterologous production of terpenoids in *C. reinhardii*. To address this, terpenoid metabolism was engineered by RNAi-mediated silencing of genes involved in competing pathways that used FPP as a substrate including a squalene synthase and geranyl-geranyl-pyrophosphate synthase. Down-regulation of these competing enzymes resulted in bisabolene yields up to 4.8 mg L^−1^. This was further improved to 11.0 mg L^−1^ by cultivating the engineered algae in mixotrophic light/dark (16 h/8 h) regimes and providing both 3% CO_2_ and acetate in the growth medium [48]. Yields were, however, significantly lower than yields obtained in yeast (more than 900 mg L^−1^) [123]. Very recently, Laursen et al. showed that chloroplast localization of diterpenes synthases is essential for the production of diterpenes casbene and manoyl oxide in *C. reinhardtii*. Further carbon flow optimization by fusing the terpene synthases to a mutated GGPP synthase resulted in the production increase to 25 mg L^−1^ manoyl oxide [84].

This work demonstrated the feasibility and efficacy of fine-tuning of isoprenoid metabolism in *C. reinhardii* to improve terpenoid yields, but it also underscored that terpenoid production is strongly influenced by cultivation conditions. Therefore, understanding the basis of the endogenous regulation of the isoprenoid pathways will be key toward designing efficient metabolic engineering and bioprocessing strategies. Nuclear transformation in *C. reinhardtii* occurs by random chromosomal integration of the transgene. Therefore, transgene expression is highly variable across independent cell lines (due to the position-of-integration effects) and may be subjected to currently poorly-understood endogenous silencing mechanisms possibly both at the transcriptional and translational level. Therefore, high-throughput screening platforms are required for identifing the best performing strains. In addition, a targeted gene integration technique would improve reproducibility.

#### 3.2.2. Diatoms

Diatoms are taxonomically distant from higher plants but, being eukaryotic like higher plants, they have both MVA and MEP pathways for isoprenoid precursor production. Diatoms are known to produce high amounts of carotenoids (some of which are high-value and exclusively produced by this group [121]), and a large variety of sterols [121]. Some are also known to emit and contribute a major proportion of marine isoprene [124]. *Thalassiosira pseudonona* and *Phaeodactylum tricornutum* have emerged in recent years as models for the two main diatom morphological sub-groups (centric and pennate).

*Phaeodactylum tricornutum* is a potentially promising candidate because of its peculiar organization of central carbon metabolism, which comprises three different glycolytic pathways. Besides the Emden Meyerof Parnas glycolysis, *P. tricornutum* possesses a putative phosphoketolase pathway and a functional eukaryotic Entner-Doudoroff pathway [113]. Although the role of the phosphoketolase pathway and Entner-Doudoroff glycolytic pathways in diatoms is not clear yet, they both produce NADPH and molecules that could possibly enter and fuel the MVA and the MEP pathways. In fact, the putative phosphoketolase pathway possibly produces acetyl-coA (and 1 ATP and NADPH) and the Entner-Doudoroff pathway yields glyceraldehyde-3-phosphate and pyruvate (and 1 ATP and NADPH). These two pathways, which are naturally present in *P. tricornutum*, have been heterologously expressed and employed by engineered yeasts—where they are absent—for increased availability of isoprenoid precursors [69].

An investigation of the genetic basis of isoprenoid metabolism in *P. tricornutum* revealed several unusual features including the absence of a conventional squalene epoxidase enzyme and the combination of the enzymatic activities of IPP isomerase and squalene synthase in a fusion enzyme [125]. These unusual characteristics likely indicate that a particular selective pressure has been exerted on these parts of the metabolism.

Diatoms benefit from the most advanced genetic toolbox among algae and the availability and the efficiency of the molecular tools to genetically manipulate diatoms has recently progressed enormously. High-efficiency genetic transformation can be achieved by several means ranging from biolistic DNA-coated particle bombardment, electroporation, and bacteria-mediated conjugation in which the latter is compatible with the transfer of large DNA constructs [111]. Methods and resources for transgene expression, RNAi-mediated silencing [126], and CRISPR/Cas9-mediated genome editing [127,128] have been established. Key to synthetic biology applications, recent advancements have shown that specific centromeric sequences can be added to large episomes to enable extrachromosomal stable maintenance [111], which theoretically allows the simultaneous transformation of very large constructs or multiple genes. This overcomes issues related to the random genomic insertion of the transgenes, which is one of the main bottlenecks in algal genetics transformation. Moreover, recent efforts have succeeded in developing high efficiency targeted genome editing techniques in diatoms [128]. Metabolic engineering efforts in diatoms have been mostly targeted to their lipid metabolism with only a handful of examples of heterologous terpenoid production. However, recent work has highlighted the potential of these organisms. For example, overexpression of the DXS enzyme in the MEP pathway of *P. tricornutum* resulted in the 2.4 fold increase in the yield (24.4 mg g^−1^ DW) of the native high-value carotenoid fucoxanthin [129], which highlights the role of DXS in controlling the flux through the MEP pathway to the carotenoid end-products. DXS is the entry point from central carbon metabolism for the MEP pathway and is the primary rate-limiting step in other organisms used for isoprenoid production.

To date, there is only one example of heterologous terpenoid production in diatoms. This was aimed at the production of plant triterpenoid saponigenin by installing a synthetic branch in the sterol biosynthesis of *P. tricornutum*. The expression of a *Lotus japonicus* lupeol synthase in *P. tricornutum* enabled the conversion of 2,3-epoxysqualene into lupeol to the maximum titre of 0.1 mg l^−1^ and the conversion of this to trace amounts of betulin by expressing a *Medicago truncatula* CYP450 oxidase (MtCYP716A12) and its reductase (Mt71CPR) [85]. The production of lupeol was accompanied by a detectable decrease in sterol content, which suggests that the substrate is a limiting factor and that the installed plant pathway competes with the endogenous biosynthesis of sterols [85].

These initial efforts in terpenoid engineering in algae clearly showed the potential and the feasibility of the production of plant terpenoids in algae while underscoring that key bottlenecks provide sufficient pools of substrates and metabolic pull from heterologous production with competing essential pathways such as that from sterol biosynthesis. This points towards the need to characterize and understand endogenous isoprenoid metabolism for efficient engineering. The feasibility of transferring large extrachromosomal genetic constructs provides the opportunity for more complex and multiplexed engineering approaches for heterologous production in diatoms, which makes this group of organisms one of the most promising candidates among photosynthetic eukaryotic microbes.

## 4. Perspectives

Even though there has been considerable progress in their metabolic engineering, heterologous production of terpenoids is far from straightforward in photosynthetic microorganisms. However, with the expansion of available hosts, engineering tools, and understanding of terpenoid metabolism regulation, increased product titre and diversity will become available. Synthetic and systems biology are expected to contribute greatly to this effort by providing more options and helping in more holistic—taking into account not just the heterologous pathway and the connected metabolic branches, but the whole metabolism—engineering.

Presently, cyanobacteria and algae are limited by small genetic engineering toolboxes compared to model heterotrophs, have a lack of knowledge about regulatory mechanisms, and have relatively slow growth. They also require non-standard equipment (light growth cabinets and photobioreactors). Production under different growth conditions (hetero/mixo/autotrophic) and light regimes (continuous light/photoperiod) has shown some promise but needs to be further evaluated. As toolboxes for manipulation develop further and our knowledge of regulatory systems expands, engineering will become easier in these organisms. Moreover, expanding our engineering efforts to more algal and cyanobacterial species such as the cyanobacterium *Synechococcus* UTEX 2973, algae members of the genus *Nannochloropsis* (Eustigmatophyceae), or more non-model species may reveal strains more suitable for high terpenoid productivities. Ultimately, photosynthetic terpenoid ‘chassis cells’ that deliver reasonable base rates, titers, and yields [130] similar to those currently used for industrial production in *E. coli* and yeast will become available.

A better understanding of flux regulatory mechanisms will improve our ability to successfully redirect carbon to heterologous pathways without impinging unduly on essential metabolic processes. These mechanisms are bound to be different than the ones in heterotrophic mechanisms, given the different origin of metabolic precursors (photosynthetic) and the more complex redox regulation. Historically, terpenoid engineering in photosynthetic microbes has only just begun. The first report was just eight years ago [97] and relatively few groups work in these organisms when compared to several decades and likely hundreds of labs worldwide working in terpenoid engineering in yeast and *E. coli*. It is probable that increasing the numbers of groups will engage with these microorganisms as tools and understanding improves. This is drawn by the benefits of CO_2_ fixation as a carbon feedstock and the potential for improved production of specific target terpenoids especially decorated plant terpenoids. Photosynthetic microorganisms may provide more production options including for the expression of more complex plant pathways.

## Figures and Tables

**Table 1:** Summary of terpenoid metabolic engineering works in photosynthetic microbes.

Terpenoid	Production Organism	Titer	Reference
isoprene	*Synechocystis* sp. PCC 6803	12.30 mg g^−1^	[72]
isoprene	*Synechococcus elongatus* PCC 7942	1.26 g L^−1^	[73]
beta-phellandrene	*Synechocystis* sp. PCC 6803	3.20 mg g^−1^	[74]
limonene	*Synechococcus* sp. PCC 7002	4 mg L^−1^	[75]
limonene	*Synechocystis* sp. PCC 6803	6.70 mg L^−1^	[76]
bisabolene	*Synechococcus* sp. PCC 7002	0.60 mg L^−1^	[75]
farnesene	*Anabaena* sp. PCC 7120	0.08 mg g^−1^	[77]
amorphadiene	*Synechococcus elongatus* PCC 7942	19.80 mg L^−1^	[78]
squalene	*Synechocystis* sp. PCC 6803	1.20 mg g^−1^	[79]
farnesene	*Synechococcus elongatus* PCC 7942	4.60 mg L^−1^	[80]
geranyllinalool	*Synechocystis* sp. PCC 6803	0.36 mg g^−1^	[81]
manoyl oxide	*Synechocystis* sp. PCC 6803	2 mg L^−1^	[82]
(E)-alpha-bisabolene	*Chlamydomonas reinhardtii*	11 mg L^−1^	[48]
patchoulol	*Chlamydomonas reinhardtii*	0.47 mg L^−1^	[83]
manoyl oxide	*Chlamydomonas reinhardtii*	50 mg L^−1^	[84]
lupeol	*Phaeodactylum tricornutum*	0.1 mg L^−1^	[85]
